# *Bifidobacterium* Mediates the Associations Between the Dietary Approaches to Stop Hypertension (DASH) Diet and Blood Pressure and Blood Lipids in Chinese Adults

**DOI:** 10.3390/nu18050797

**Published:** 2026-02-28

**Authors:** Qiong Zhang, Yun Zhang, Maoxin Ren, Yanjun Deng, Yuanyao Chen, Guang Li, Hao Feng, Xiaobao Wang, Yuhao Cui, Jiamei Huang, Yixuan Xu, Xiaomin Li, Sumei Xiao

**Affiliations:** 1 Department of Epidemiology, School of Public Health, Sun Yat-sen University, Guangzhou 510080, China; zhangq593@mail2.sysu.edu.cn (Q.Z.); zhangy2675@mail2.sysu.edu.cn (Y.Z.); renmx3@mail2.sysu.edu.cn (M.R.); dengyj66@alumni.sysu.edu.cn (Y.D.); liguang8@alumni.sysu.edu.cn (G.L.); fenghao_chn@163.com (H.F.); wangxiaobao56@163.com (X.W.); cuiyh9@mail2.sysu.edu.cn (Y.C.); huangjm83@alumni.sysu.edu.cn (J.H.); xuyx58@mail2.sysu.edu.cn (Y.X.); 2 Research & Development Division, Perfect Life & Health Institute, Zhongshan 528400, China; yuanyaochen0401@163.com; 3 School of Chinese Materia Medica, Shenyang Pharmaceutical University, Shenyang 110016, China; 4 Guangdong Provincial Key Laboratory of Food, Nutrition and Health, School of Public Health, Sun Yat-sen University, Guangzhou 510080, China

**Keywords:** DASH, blood pressure, blood lipids, gut microbiota, *Bifidobacterium*

## Abstract

**Background**: The Dietary Approaches to Stop Hypertension (DASH) diet effectively lowers blood pressure and improves blood lipid profiles. However, it remains unclear how the DASH diet contributes to gut microbiota and how the gut microbes affect these processes. This study aimed to examine the associations of DASH diet adherence with blood pressure and lipid levels, and to explore whether the gut microbiota mediated these relationships. **Methods**: A total of 879 Chinese aged over 18 years were recruited. DASH diet adherence was evaluated by a food frequency questionnaire. Blood pressure and lipid measurements were obtained during physical examinations. The gut microbiota was analysed via 16S rRNA sequencing. **Results**: Greater DASH diet adherence was correlated with lower diastolic blood pressure (DBP; sβ = −0.180 and *p* = 0.027) and low-density lipoprotein cholesterol (LDL-C; sβ = −0.268 and *p* = 0.002). Five bacterial genera were associated with the DASH diet (*q*-value < 0.15). Among them, *Bifidobacterium* was inversely linked to both DBP and LDL-C (*p* < 0.050). Two species (*Bifidobacterium kashiwanohense* and *Bifidobacterium longum*) were detected within the *Bifidobacterium* genus. Both of them explained the associations of the DASH diet with DBP and/or LDL-C (average causal mediation effect = −0.027 to −0.018; *p* < 0.050; proportion mediated = 8.22% to 9.04%). **Conclusions**: This study found favourable correlations of the DASH diet with both DBP and LDL-C. *Bifidobacterium* partially explained these relationships. These results may offer valuable insights into managing blood pressure and lipid levels through dietary and gut microbiota modulation.

## 1. Introduction

Cardiovascular disorders (CVDs) are the major cause of premature mortality. Hypertension and dyslipidaemia are important risk factors for CVDs [1]. Adopting a healthful dietary pattern, such as the Dietary Approaches to Stop Hypertension (DASH) diet, may effectively ameliorate these conditions and help prevent the onset and progression of CVDs. The DASH diet emphasises intakes of whole grains, fruits, vegetables, fat-free or low-fat dairy products, nuts, and legumes, while limiting red and processed meat, total fat, saturated fat, cholesterol, sweets, added sugars, and sugar-sweetened beverages. Previous studies consistently reported that the DASH diet decreased blood pressure [2]. A quantitative systematic review of randomised clinical trials (RCTs) also reported that it significantly lowered total cholesterol (TC), low-density lipoprotein cholesterol (LDL-C), and very-low-density lipoprotein cholesterol [3].

Gut microbes hold crucial regulatory roles in blood pressure and lipid metabolism. They influence them via multiple mechanisms, such as regulation of immune responses, inflammation, and energy metabolism [4,5]. A study of 6953 Finnish individuals found that 45 genera were detrimentally correlated with blood pressure and 19 genera were favourably correlated [6]. Mendelian randomisation studies revealed several causal relationships: the *Clostridium innocuum* group, *Eubacterium fissicatena* group, *Lachnospiraceae FCS020* group, and *Olsenella* were related to a higher risk of hypertension, and *Flavonifractor*, *Parabacteroides*, and *Senegalimassilia* were related to a lower risk [7]. Faecal microbiota transplantation was also revealed to reduce systolic blood pressure (SBP) relative to the placebo group [8]. For blood lipids, a 27-year cohort study of 10,678 Chinese individuals found that several genera (*Turicibacter*, *Clostridium sensu stricto 1*, *CHKCI002*, *[Eubacterium] brachy group*, and *Parabacteroides*) were linked to a lower risk of dyslipidaemia [9]. Mendelian randomisation studies indicated that higher relative abundances of *Alistipes* and *Oscillibacter* were causally associated with reduced triglyceride (TG) concentrations [10]. In addition, an RCT study demonstrated that supplementation with *Bifidobacterium lactis* IDCC 4301 for 12 weeks significantly reduced serum TG concentrations [11].

The gut microbiota may, therefore, mediate the association of diet with blood pressure and lipids. Previous studies showed that dietary patterns may affect the composition and function of the intestinal microbiome. For example, healthy plant-based diet adherence was observed to be positively linked to the relative abundances of three polysaccharide-degrading bacteria (*Bacteroides thetaiotaomicron*, *Eubacterium eligens*, and *Faecalibacterium prausnitzii*) and six other genera (*Blautia hydrogenotrophica*, *Alistipes shahii*, *Sellimonas ingoalis*, *Eisenbergiella massiliensis*, *Blautia wexlerae*, and *Dorea* sp. *CAG 317*) [12]. RCT studies also demonstrated the alteration of gut microbiota composition after dietary interventions. After four weeks of Mediterranean diet intervention (*n* = 82), the relative abundances of *Streptococcus thermophilus, Ruthenibacterium lactatiformans*, *Ruminococcus gnavus*, *Ruminococcus torques*, *Parabacteroides merdae*, and *Flavonifractor plautii* were decreased, while *Faecalibacterium prausnitzii* was enriched [13]. Recently, a six-month RCT study in pre-diabetic individuals (*n* = 200) reported that *Bacteroidales*, *Lachnospiraceae*, and *Oscillospirales* mediated the beneficial impacts of a personalised postprandial-targeting diet on cardiometabolic outcomes, including haemoglobin A1c, TG, and high-density lipoprotein cholesterol (HDL-C) [14]. These results indicated that gut microbes may act as a mediator in the correlation of the DASH diet with blood pressure and lipid metabolism.

To date, it remains unclear how the DASH diet influences the gut microbiome’s diversity and composition, and how gut microbes affect the correlation of the DASH diet with blood pressure and lipids. This study, therefore, aimed to investigate the correlations of DASH diet adherence with blood pressure and lipids in a Chinese population, as well as to explore the potential mediating role of gut microbes in these relationships. These findings may offer some valuable insights into managing blood pressure and lipid levels via dietary intervention and gut microbiota regulation, and may contribute to elucidating the pathogenesis of hypertension and dyslipidaemia.

## 2. Materials and Methods

### 2.1. Study Participants

In this cross-sectional study, a total of 1012 Chinese individuals aged over 18 years were recruited between May 2023 and May 2024 through advertisements, community leaflet distribution, and social media. They all lived in Guangdong for at least one year. Individuals were excluded due to (1) having severe illnesses, such as malignant diseases; (2) using antihypertensive or lipid-lowering medications; (3) having severe gastrointestinal disorders, such as ulcerative colitis or irritable bowel syndrome; (4) being pregnant, lactating, or preparing for pregnancy; (5) taking probiotic supplements or antibiotics over the last month; (6) having abnormal total energy intake (<600 or >3500 kcal/day for females; <800 or >4200 kcal/day for males); and (7) having incomplete information of dietary intake or missing blood pressure or lipid data. Finally, 879 participants were enrolled in the statistical analyses. This study was approved by the Institutional Ethics Committee of the School of Public Health, Sun Yat-sen University (approval number: 2023-61; approval date: 11 April 2023). Informed consent was acquired in writing from each participant.

### 2.2. Assessment of the DASH Diet

A validated and reliable food frequency questionnaire (FFQ) was adopted to estimate dietary intake within the past 12 months [15]. A face-to-face interview was performed to gather the relevant information by trained investigators. The FFQ included the most commonly consumed foods in Guangdong Province, with eight food groups and one item on dietary salt. For each food item, frequency (never, per day, per week, per month, or per year) and portion size were estimated. Food models and photographs depicting standard portion sizes were adopted to assist the individuals in assessing their dietary intakes. Intake of total energy was computed using the data from the Chinese Food Composition Table (6th ed.) [16]. DASH diet adherence was evaluated following the method proposed by Fung et al. [17]. This scoring system includes eight components: whole grains, vegetables, fruits, legumes and nuts, dairy products, sugar-sweetened beverages, sodium, and red or processed meat. As low-fat dairy product consumption in the target population was relatively low, this component was replaced with regular dairy products [18]. Participants were classified into quintiles based on their average daily intake of each component. For favourable components (whole grains, vegetables, fruits, legumes and nuts, and dairy products), scores of 1–5 were assigned from the lowest to the highest quintile. For unfavourable components (sugar-sweetened beverages, sodium, and red or processed meat), the scoring was reversed (5–1). The total DASH score (range: 8–40) was computed as the sum of all component scores and further divided into tertiles for the subsequent analyses.

### 2.3. Assessment of Blood Pressure and Blood Lipids

SBP and diastolic blood pressure (DBP) were evaluated twice at one-minute intervals following a 2–5 min seated rest, utilising an automatic sphygmomanometer (OMRON HBP-9020; Omron Healthcare (China) Co., Ltd., Dalian, China). Venous blood samples were collected following an overnight fast lasting no less than 12 h. Serum was separated by centrifugation at 3000 rpm for 10 min at 4 °C. Six lipid measurements, such as TC, TG, HDL-C, LDL-C, apolipoprotein A (ApoA) and apolipoprotein B (ApoB), were tested by an automatic biochemical analyser (Atellica CH930; Siemens Healthineers, Erlangen, Germany).

### 2.4. Measurement of Other Variables

Sociodemographic data (age and sex) and lifestyle factors (drinking, smoking and physical activity) were collected via a self-administered questionnaire. Drinking and smoking were binary variables (no/yes). Drinking referred to alcohol consumption at least once weekly for no less than six consecutive months. Smoking referred to consuming more than 100 cigarettes over a lifetime. Physical activity was evaluated by the long version of the International Physical Activity Questionnaire (IPAQ-L) and expressed in metabolic equivalent hours per day (MET.h/d) [19]. Height was assessed to the nearest 0.1 cm utilising a Harpenden stadiometer (Holtain Ltd., Crymych, Wales, UK). Weight was recorded to the nearest 0.1 kg by an Inbody 770 analyser (InBody Co., Ltd., Seoul, Republic of Korea). Body mass index (BMI) was computed as weight (kg) divided by height squared (m^2^). Four BMI categories [20] were defined as underweight (<18.5 kg/m^2^), normal weight (18.5 to <24.0 kg/m^2^), overweight (24.0 to <28.0 kg/m^2^) and obese (≥28.0 kg/m^2^).

### 2.5. Faecal DNA Extraction and 16S rRNA Sequencing

Each participant was provided with a stool collection kit and instructed to collect fresh faecal samples (≥1 g). Samples were dispensed into aliquots within 4 h, snap-frozen in liquid nitrogen for 30 min, and preserved at −80 °C. Faecal DNA was extracted utilising the MagaBio Soil and Feces Genomic DNA Purification Kit (Bioer, Hangzhou, China). Amplification of the V3–V4 hypervariable region of the 16S rRNA gene was performed with the primers 341F (5′-CCTACGGGNGGCWGCAG-3′) and 785R (5′-GACTACHVGGGTATCTAATCC-3′). The PCR products were purified with the AxyPrep DNA Gel Extraction Kit (Axygen Biosciences, Union City, CA, USA) and subsequently quantified via QuantiFluor-ST™ (Promega, Madison, WI, USA). Sequencing was conducted by the Illumina MiSeq PE300 platform (Illumina, San Diego, CA, USA). Raw paired-end sequencing data were processed with the EasyAmplicon pipeline (v1.21) [21]. VSEARCH (v2.22.1) was adopted to merge reads, trim primers, filter low-quality sequences and dereplicate. Clean tag was clustered into operational taxonomic units (OTUs) using USEARCH (v11.0.667) at 97% sequence similarity. Chimaeras were removed using VSEARCH with the SILVA database (v123), and an OTU table was constructed. Taxonomic annotations for each OTU were acquired by analysing the microbial community composition of each sample across all taxonomic ranks (domain, phylum, class, order, family, genus, and species).

### 2.6. Statistical Analysis

For the variables of basic characteristics, the continuous variables were expressed as means (standard deviation (SD)) and medians (interquartile range (IQR)), and the categorical variables were expressed as frequencies (percentage). A multivariate linear regression model was adopted to test the relationships of the DASH diet with blood pressure and lipids. Two models were constructed: model 1 adjusted for age, sex, and BMI; model 2 additionally adjusted for smoking, drinking, physical activity and total energy intake.

For gut microbiota analyses, α-diversity indices, namely, Shannon, Simpson, richness, and Chao1, were computed. The comparison of α-diversity across DASH tertiles was carried out with one-way analysis of variance followed by Tukey’s honest significant difference test. Principal coordinates analysis based on Bray–Curtis dissimilarities was conducted to visualise the β-diversity among the tertiles. In addition, the permutational multivariate analysis of variance with 999 permutations was conducted to evaluate the overall microbial composition across tertiles at the genus level. The multivariate association with linear models (MaAsLin) was adopted to detect the DASH diet-associated differential microbial genera, with a significance threshold set at a *q*-value of less than 0.150. The Benjamini–Hochberg method was used to compute the adjusted *p*-value to control the false discovery rate (FDR). Furthermore, the linear regression analyses were utilised to assess the relationships of DASH-related differential genera with blood pressure and lipid levels. Overlapping genera associated with both the DASH diet and blood pressure/lipids were identified as microbial biomarkers. To quantify the gut microbial features, a microbial index (MI) was calculated using the relative abundances of these biomarkers. MI = ∑(+/− relative abundance of microbial biomarkers; +/− is the direction of the correlation between the DASH diet and gut microbial biomarkers). Mediation analyses were carried out with the ‘mediate’ function of the mediation package in R, to explore the mediating effect of gut microbes on the associations between the DASH diet and blood pressure/lipids. The average causal mediation effect (ACME), average direct effect (ADE), the proportion mediated, and their 95% confidence intervals (95% CI) were assessed via quasi-Bayesian Monte Carlo simulation with 2000 iterations. The confounders, such as age, sex, BMI, smoking, drinking, physical activity, and total energy intake, were adjusted in all analyses. R software (v4.4.1) was applied, and a two-sided *p*-value of less than 0.05 was considered statistically significant.

## 3. Results

### 3.1. Characteristics of the Study Participants

Table 1 shows the basic characteristics of the study participants. Of the included 879 individuals, 66.4% were females, 8.8% were smokers, and 11.7% were drinkers. The median (IQR) age was 37.0 (25.0–48.0) years. The mean (SD) BMI was 22.52 (3.18) kg/m^2^. The median (IQR) daily physical activity was 23.80 (18.24–30.45) MET·h/d. The median (IQR) total energy intake and DASH score were 1540 (1216–1880) kcal/day and 24 (21–27), respectively. The median (IQR) systolic and diastolic blood pressures were 110.00 (101.00–120.00) mmHg and 68.00 (61.75–75.00) mmHg, respectively. The median (IQR) values of lipid parameters were as follows: TG 1.02 (0.76–1.44) mmol/L, TC 4.83 (4.29–5.54) mmol/L, LDL-C 2.70 (2.21–3.19) mmol/L, HDL-C 1.36 (1.12–1.62) mmol/L, ApoA 1.35 (1.19–1.54) g/L and ApoB 0.90 (0.73–1.08) g/L.

### 3.2. Associations of the DASH Diet with Blood Pressure/Lipids

Table 2 shows the correlations of DASH diet adherence with blood pressure/lipid parameters. Participants were divided into tertiles according to their total DASH scores, with the lowest tertile (T1) as the reference group. In model 2, after the adjustment of age, sex, BMI, smoking, drinking, physical activity, and total energy intake, the highest DASH score tertile (T3) had the lowest DBP (sβ = −0.180; 95% CI: −0.339 to −0.021; *p* = 0.027) and the lowest LDL-C (sβ = −0.268; 95% CI: −0.435 to −0.101; *p* = 0.002). In addition, the linear trend tests across the three DASH tertiles were significant for these associations (*P*_-trend_ = 0.028 for DBP and *P*_-trend_ = 0.002 for LDL-C).

### 3.3. Gut Microbiota Characteristics Related to the DASH Diet

**Figure 1:**
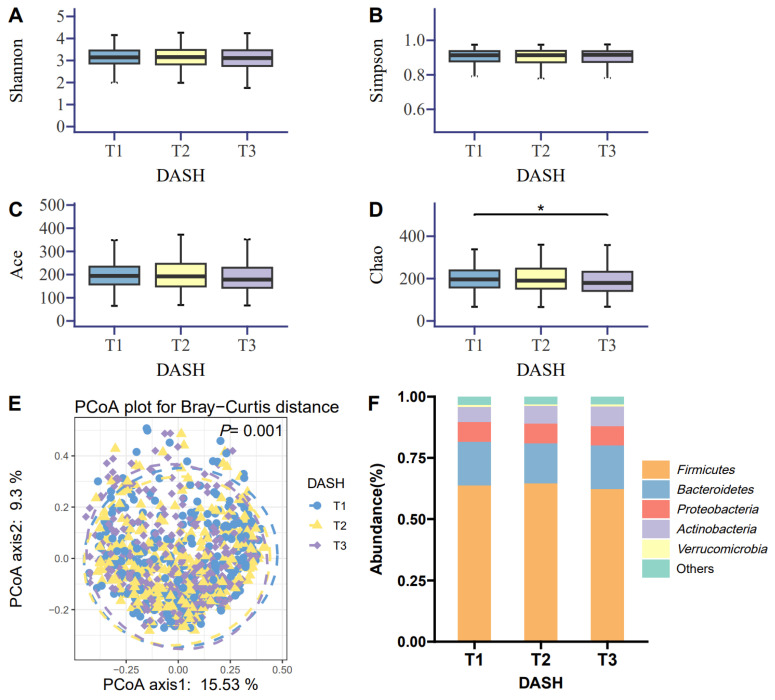
Associations between DASH tertiles and gut microbiota. (**A**–**D**) α-diversity indices, including Shannon, Simpson, Ace and Chao across DASH tertiles. (**E**) β-diversity among DASH tertiles visualised via a principal coordinates analysis plot based on Bray–Curtis dissimilarities at the genus level. (**F**) Taxonomic distribution of the five most abundant bacterial phyla across DASH tertiles. DASH, Dietary Approaches to Stop Hypertension. *, *p* < 0.050.

Among the α-diversity indices, the Chao index showed a significant difference between the highest (T3) and lowest (T1) DASH tertiles (*p* < 0.050; Figure 1D). No significant difference was shown in the Simpson, Shannon, and Ace indices across DASH tertiles (all *p* > 0.050; Figure 1A–C). Regarding β-diversity, principal coordinates analysis based on the Bray–Curtis distance at the genus level revealed significant differences among the three DASH tertiles (*p* = 0.001; Figure 1E). The five most abundant taxa were *Firmicutes*, *Bacteroidota*, *Proteobacteria*, *Actinobacteria*, and *Verrucomicrobia* at the phylum level (Figure 1F).

### 3.4. Differential Gut Microbial Genera Associated with Both the DASH Diet and Blood Pressure/Lipids

**Figure 2:**
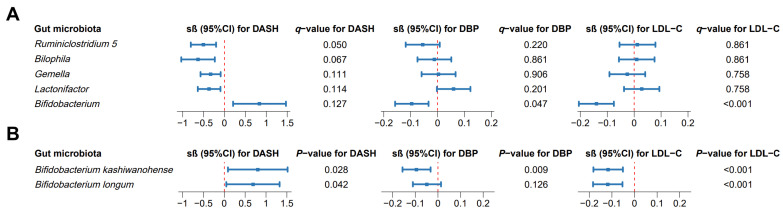
Differential gut microbiota associated with both the DASH diet and blood pressure and lipid parameters. (**A**) Association results of differential gut microbes at genus level; (**B**) association results of *Bifidobacterium* at species level. DASH, Dietary Approaches to Stop Hypertension; DBP, diastolic blood pressure; LDL-C, low-density lipoprotein cholesterol.

As shown in Figure 2A, five differential microbial genera were identified between the highest (T3) and lowest (T1) DASH tertiles after adjusting for the confounders (*q*-value < 0.150). Among them, *Ruminiclostridium 5*, *Bilophila*, *Gemella* and *Lactonifactor* were negatively associated and *Bifidobacterium* was positively associated with the DASH diet, respectively. Among these five differential genera, *Bifidobacterium* also showed negative associations with DBP (sβ = −0.095 and *q*-value = 0.047; Figure 2A) and LDL-C (sβ = −0.140 and *q*-value < 0.001; Figure 2A) after the adjustment of confounders (*n* = 879).

In these 16S rRNA sequencing data, two species with a prevalence above 10% were detected, namely, *Bifidobacterium kashiwanohense* and *Bifidobacterium longum*, within the *Bifidobacterium* genus. Both species were positively associated with the DASH diet after adjusting for the confounders (*p* < 0.050; Figure 2B). *Bifidobacterium kashiwanohense* was inversely linked to DBP (sβ = −0.094; *p* = 0.009) and LDL-C (sβ = −0.117; *p* < 0.001, Figure 2B). *Bifidobacterium longum* had a negative correlation with LDL-C (sβ = −0.118 and *p* < 0.001; Figure 2B).

### 3.5. Mediating Roles of Bifidobacterium in the Relationships of the DASH Diet with Blood Pressure/Lipids

**Figure 3:**
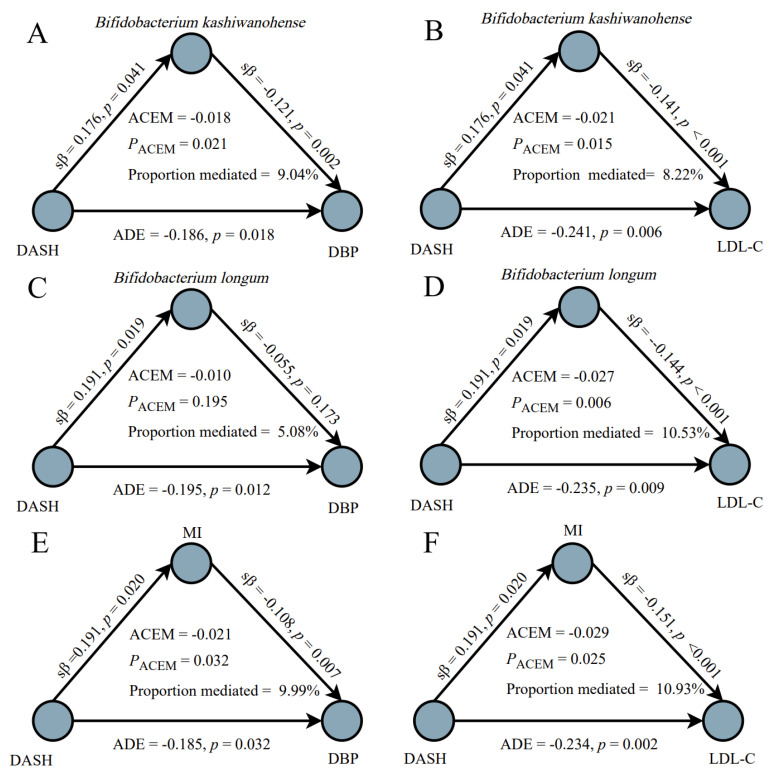
Mediation of the relationships between the DASH diet and blood pressure and lipids by identified differential gut microbes (**A**–**D**) and microbial index (MI, (**E**,**F**)). DASH, Dietary Approaches to Stop Hypertension; DBP, diastolic blood pressure; LDL-C, low-density lipoprotein cholesterol; ACME, the average causal mediation effect; ADE, the average direct effect.

The mediation analysis results are shown in Figure 3. After adjusting the confounders, at the species level, *Bifidobacterium kashiwanohense* mediated the associations of the DASH diet with both DBP (ACME = −0.018, *p* = 0.021, and proportion mediated = 9.04%; Figure 3A) and LDL-C (ACME = −0.021, *p* = 0.015, and proportion mediated = 8.22%; Figure 3B). *Bifidobacterium longum* mediated the association between the DASH diet and LDL-C (ACME = −0.027, *p* = 0.006, and proportion = 10.53%; Figure 3D). Furthermore, the MI index combining these two species also showed the mediation role in the relationships of the DASH diet with DBP (ACME = −0.021, *p* = 0.032, and proportion mediated = 9.99%; Figure 3E) and LDL-C (ACME = −0.029, *p* = 0.025, and proportion mediated = 10.93%; Figure 3F). Reverse mediation analyses were then conducted, and no significant mediating effects were observed (all *p* > 0.050; Supplementary Figure S1).

## 4. Discussion

In this study, the correlations of the DASH diet with blood pressure and lipids, as well as the mediating roles of gut microbes, were detected in 879 Chinese adults. The results revealed that the DASH diet was negatively linked to both DBP and LDL-C. Five differential bacterial genera were related to the DASH diet. Among them, *Bifidobacterium* was also correlated with DBP and LDL-C. Furthermore, two species (*Bifidobacterium kashiwanohense* and *Bifidobacterium longum*) identified within this genus, and their combined MI, were observed to partially explain the associations of the DASH diet with DBP and LDL-C.

The DASH diet showed beneficial associations with DBP and LDL-C. These results were consistent with previous studies. A quantitative systematic review of 30 RCTs involving 5545 participants indicated that the DASH diet lowered DBP by 2.5 mmHg relative to the control group [22]. For LDL-C, a quantitative systematic review conducted in 2025, including 22 RCTs with 3562 overweight and obese individuals, found that the DASH diet decreased LDL-C levels by 5.33 mg/dL [3]. The health benefits of the DASH diet are largely attributable to its favourable nutritional quality and composition. As it is rich in vegetables and fruits, the DASH diet increases intakes of potassium and dietary fibre. Low potassium intake increases DBP by promoting renal sodium retention and raising salt sensitivity [23]. Dietary fibre may undergo fermentation by gut microbes to produce short-chain fatty acids (SCFAs), among which butyrate may exert favourable regulatory effects on DBP via modulating water–sodium homeostasis, hormonal signalling systems, and anti-inflammatory pathways [24]. Additionally, SCFAs were shown to modulate LDL-C metabolism through the activation of SCFA receptors on the liver and adipose tissue [25]. Meanwhile, dietary fibre enhances the excretion of cholesterol via faeces, thereby reducing LDL-C levels [26].

This study revealed that DASH diet adherence was correlated with five differential gut bacterial genera. Previous studies supported the observed relationships between these genera and dietary patterns. In a one-year RCT study with 343 overweight and obese individuals, the abundances of *Ruminiclostridium 5* and *Bilophila* were lower in the intervention group receiving an energy-restricted Mediterranean diet combined with physical activity promotion [27]. Data from 21,561 individuals indicated that compared with vegetarians, *Bilophila* was a signature microbiota of omnivores and was positively associated with red meat intake [28]. For *Gemella*, its abundance was increased in mice fed a high-fat diet supplemented with lard [29]. *Lactonifactor* was also reported to be positively related to dietary animal protein intake [30]. Notably, both the Mediterranean diet and vegetarian dietary patterns were reported to be positively associated with *Bifidobacterium* abundances [31,32,33]. It is one of the most widely used probiotic genera for maintaining human gut health [34].

The relationships of the DASH diet with DBP and LDL-C could be partially explained by the *Bifidobacterium* genus. Previous studies also indicated the favourable effect of *Bifidobacterium* on blood pressure and lipid metabolism. In an RCT (*n* = 210) study, oat intervention significantly increased *Bifidobacterium* abundance, which was negatively correlated with LDL-C [35]. Another study reported that *Bifidobacterium* was enriched in the controls, compared with the hypertension patients [36]. As for specific species of *Bifidobacterium*, an RCT (*n* = 60) study showed that intervention with heat-treated *Bifidobacterium longum CECT 7347* lowered TC and non-HDL cholesterol [37]. Animal studies further revealed that *Bifidobacterium longum B-53* was more effective than metformin in decreasing LDL-C, TC, and TG levels, exerting the lipid-lowering effects synergistically through metabolic regulation, inflammation inhibition and gut microbiota remodelling [38]. Although no study has directly explored the associations of *Bifidobacterium kashiwanohense* with blood pressure and lipid levels, a study in Saudi women found a negative correlation between *Bifidobacterium kashiwanohense* and insulin resistance [39]. In addition, a Mendelian randomisation study revealed a negative correlation of *Bifidobacterium kashiwanohense* with colorectal cancer risk [40]. These studies supported the finding that *Bifidobacterium* acts as a mediator in the association of the DASH diet with blood pressure and lipids in this study.

To the best of our knowledge, this was the first study to reveal the DASH diet-related gut microbes and observed that *Bifidobacterium* partially explained the correlation of the DASH diet with blood pressure and lipid parameters. However, there are several limitations to this study. First, dietary intake assessed by FFQ is subject to recall bias. The FFQ employed herein exhibited good reliability and validity relative to six 3-day dietary records [15]. Food models and photographs were also used to depict the standard portions in the investigation. Second, 16S rRNA sequencing was adopted. Although this approach allows for the characterisation of microbial community structures, it is constrained by insufficient taxonomic resolution. Future studies are warranted using shotgun metagenomic sequencing to further clarify how the DASH diet and different species or strains of *Bifidobacterium* regulate blood pressure and lipids. Third, residual confounding cannot be excluded, even though key confounders were accounted for in the analyses. Moreover, the study subjects were recruited exclusively from Guangdong Province, which may restrict the generalisability of the results to other populations. Finally, due to the cross-sectional design, the causal relationship among the DASH diet, gut microbiota, and blood pressure and lipids cannot be established. The findings, therefore, warrant further exploration via longitudinal and experimental studies.

## 5. Conclusions

In conclusion, this study observed the favourable correlations of the DASH diet with DBP and LDL-C. Among the five DASH diet-related gut bacterial genera, *Bifidobacterium* partially explained these associations. These findings may offer valuable insights into the management of blood pressure and lipids by modulating dietary and gut microbiota.

## Figures and Tables

**Table 1:** Basic characteristics of the study participants (*n* = 879).

Variables	Mean/Median/n	SD/IQR/Percentage
Age (years) ^b^	37.0	25.0–48.0
Female (*n*, %) ^c^	584	66.4
BMI (kg/m^2^) ^a^	22.52	3.18
Underweight (*n*, %) ^c^	72	8.19
Normal weight (*n*, %) ^c^	536	60.87
Overweight (*n*, %) ^c^	220	25.03
Obesity (*n*, %) ^c^	51	5.80
Smoking (*n*, %) ^c^	77	8.8
Drinking (*n*, %) ^c^	103	11.7
Physical activity (MET.h/d) ^b^	23.80	18.24–30.45
Total energy intake (kcal/day) ^b^	1540	1216–1880
DASH score ^b^	24	21–27
SBP (mmHg) ^b^	110.00	101.00–120.00
DBP (mmHg) ^b^	68.00	61.75–75.00
TG (mmol/L) ^b^	1.02	0.76–1.44
TC (mmol/L) ^b^	4.83	4.29–5.54
LDL-C (mmol/L) ^b^	2.70	2.21–3.19
HDL-C (mmol/L) ^b^	1.36	1.12–1.62
ApoA (g/L) ^b^	1.35	1.19–1.54
ApoB (g/L) ^b^	0.90	0.73–1.08

**Notes**: ^a^, values are expressed as means ± standard deviation (SD); ^b^, values are expressed as medians (interquartile range (IQR)); ^c^, values are expressed as numbers (*n*) and percentages (%). Physical activity was quantified as metabolic equivalent hours per day (MET.h/d). BMI, body mass index; DASH, Dietary Approaches to Stop Hypertension; SBP, systolic blood pressure; DBP, diastolic blood pressure; TG, triglyceride; TC, total cholesterol; LDL-C, low-density lipoprotein cholesterol; HDL-C, high-density lipoprotein cholesterol; ApoA, apolipoprotein A; ApoB, apolipoprotein B.

**Table 2:** The associations of the DASH diet with blood pressure/lipids (*n* = 879).

Variable	sβ (95%CI)			*P*_-trend_
	T1	T2	T3	
SBP				
Model 1	Ref.	0.045 (−0.100~0.190)	−0.003 (−0.153~0.148)	0.976
Model 2	Ref.	0.044 (−0.102~0.190)	0.009 (−0.144~0.161)	0.902
DBP				
Model 1	Ref.	−0.037 (−0.190~0.116)	−0.204 (−0.363~−0.045) *	**0.012**
Model 2	Ref.	−0.049 (−0.201~0.103)	−0.180 (−0.339~−0.021) *	**0.028**
TG				
Model 1	Ref.	−0.090 (−0.244~0.063)	−0.100 (−0.260~0.059)	0.216
Model 2	Ref.	−0.096 (−0.251~0.058)	−0.100 (−0.262~0.062)	0.222
TC				
Model 1	Ref.	0.050 (−0.108~0.207)	−0.115 (−0.279~0.049)	0.172
Model 2	Ref.	0.040 (−0.119~0.198)	−0.117 (−0.283~0.049)	0.176
LDL-C				
Model 1	Ref.	0.017 (−0.143~0.177)	−0.277 (−0.444~−0.111) *	**0.001**
Model 2	Ref.	0.004 (−0.155~0.164)	−0.268 (−0.435~−0.101) *	**0.002**
HDL-C				
Model 1	Ref.	0.155 (0.011~0.300)	−0.109 (−0.259~0.042)	0.165
Model 2	Ref.	0.140 (−0.003~0.284)	−0.088 (−0.238~0.063)	0.277
ApoA				
Model 1	Ref.	0.116 (−0.037~0.270)	−0.103 (−0.262~0.056)	0.212
Model 2	Ref.	0.103 (−0.049~0.255)	−0.080 (−0.240~0.079)	0.343
ApoB				
Model 1	Ref.	−0.060 (−0.206~0.085)	−0.145 (−0.297~0.006)	0.060
Model 2	Ref.	−0.056 (−0.202~0.091)	−0.147 (−0.301~0.006)	0.060

**Notes**: In the multivariate linear regression analyses, two models were constructed: model 1 controlled for age, sex and BMI; model 2 further controlled for smoking, drinking, physical activity, and total energy intake; SBP, systolic blood pressure; DBP, diastolic blood pressure; TG, triglyceride; TC, total cholesterol; LDL-C, low-density lipoprotein cholesterol; HDL-C, high-density lipoprotein cholesterol; ApoA, apolipoprotein A; ApoB, apolipoprotein B. Bold, *P*_-trend_ < 0.050; *, *p* < 0.050.

## Data Availability

The original contributions presented in this study are included in this article; further inquiries can be directed to the corresponding author.

## Supplementary Materials

The following supporting information can be downloaded at https://www.mdpi.com/article/10.3390/nu18050797/s1. Figure S1. Reverse mediation of the relationship between blood pressure and lipids and the DASH diet by identified differential gut microbes (A–D) and microbial index (MI, E-F). DASH, Dietary Approaches to Stop Hypertension; DBP, diastolic blood pressure; LDL-C, low-density lipoprotein cholesterol; ACME, the average causal mediation effect; ADE, the average direct effect.
