# Supplementary material for: Bifidobacterium Mediates the Associations Between the Dietary Approaches to Stop Hypertension (DASH) Diet and Blood Pressure and Blood Lipids in Chinese Adults
Source: Nutrients. 2026 Feb 28;18(5):797. doi: 10.3390/nu18050797 (PMC12986857; doi:10.3390/nu18050797)
Supplement: Supplementary file 1 [file nutrients-18-00797-s001.zip › nutrients-4140634-supplementary.pdf]

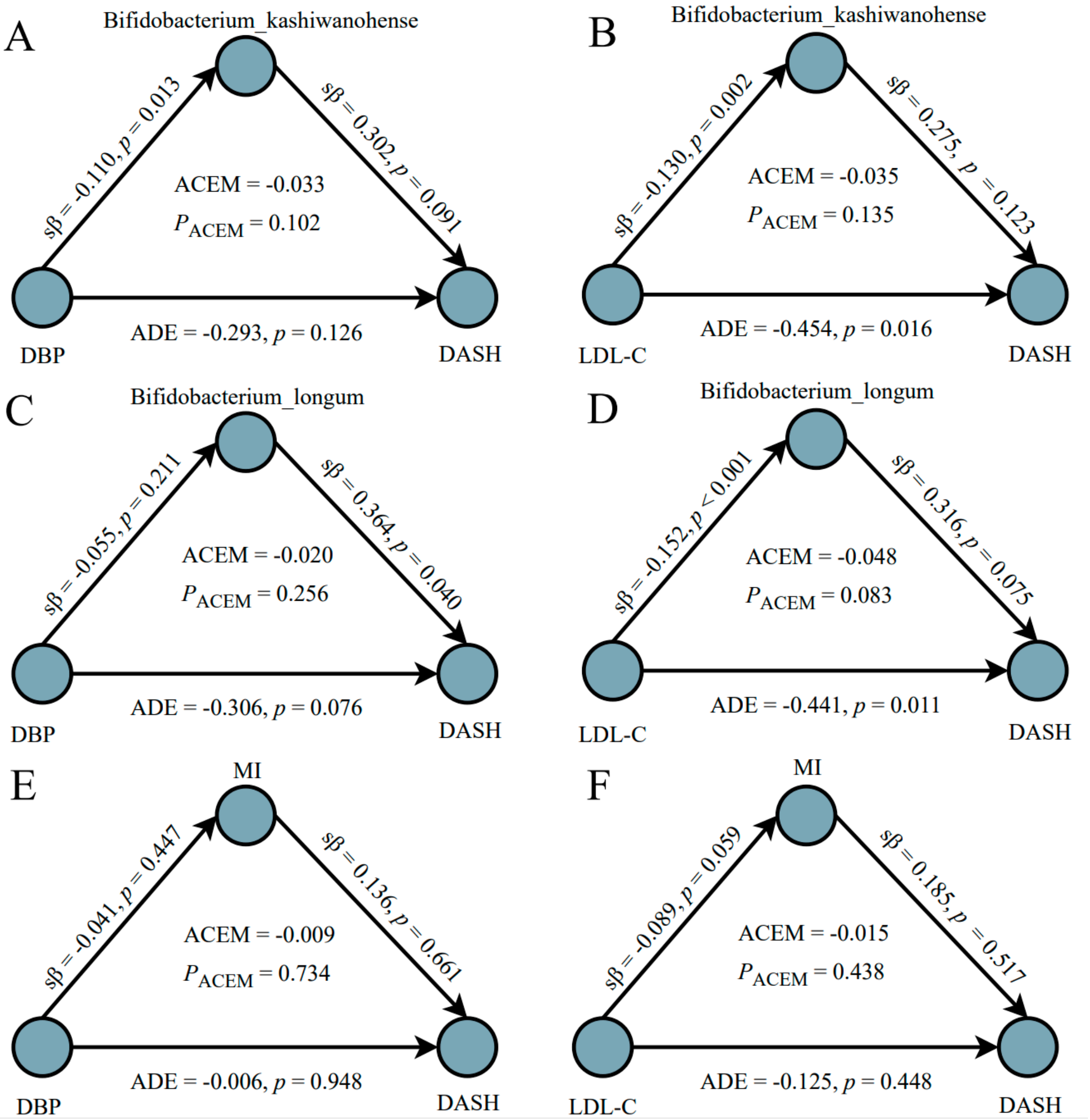

Figure S1. Reverse mediation of the relationship between blood pressure and lipids and the DASH diet by identified differential gut microbes (A-D) and microbial index (MI, E-F). DASH, Dietary Approaches to Stop Hypertension; DBP, diastolic blood pressure; LDL-C, low-density lipoprotein cholesterol; ACME, the average causal mediation effect; ADE, the average direct effect.
